# Structural insights into the regulation of *Bacillus subtilis* SigW activity by anti-sigma RsiW

**DOI:** 10.1371/journal.pone.0174284

**Published:** 2017-03-20

**Authors:** Shankar Raj Devkota, Eunju Kwon, Sung Chul Ha, Hyeun Wook Chang, Dong Young Kim

**Affiliations:** 1 College of Pharmacy, Yeungnam University, Gyeongsan, Gyeongbuk, South Korea; 2 Pohang Accelerator Laboratory, Pohang University of Science and Technology, Pohang, Gyeongbuk, South Korea; University of Washington, UNITED STATES

## Abstract

*Bacillus subtilis* SigW is localized to the cell membrane and is inactivated by the tight interaction with anti-sigma RsiW under normal growth conditions. Whereas SigW is discharged from RsiW binding and thus initiates the transcription of its regulon under diverse stress conditions such as antibiotics and alkaline shock. The release and activation of SigW in response to extracytoplasmic signals is induced by the regulated intramembrane proteolysis of RsiW. As a ZAS (Zinc-containing anti-sigma) family protein, RsiW has a CHCC zinc binding motif, which implies that its anti-sigma activity may be regulated by the state of zinc coordination in addition to the proteolytic cleavage of RsiW. To understand the regulation mode of SigW activity by RsiW, we determined the crystal structures of SigW in complex with the cytoplasmic domain of RsiW, and compared the conformation of the CHCC motif in the reduced/zinc binding and the oxidized states. The structures revealed that RsiW inhibits the promoter binding of SigW by interacting with the surface groove of SigW. The interaction between SigW and RsiW is not disrupted by the oxidation of the CHCC motif in RsiW, suggesting that SigW activity might not be regulated by the zinc coordination states of the CHCC motif.

## Introduction

In bacteria, gene transcription is initiated by sigma factor that mediates the recruitment of core RNA polymerase to a promoter region [1]. Almost all bacteria harbor multiple sigma factors, including house-keeping and additional alternative sigma factors, each of which controls the transcription of its own regulon. While a housekeeping sigma factor regulates the expression of the majority genes for bacterial homeostasis, alternative sigma factors are suppressed to a basal level by a corresponding anti-sigma factor under normal growth conditions [2]. Each alternative sigma factor is activated in response to a cognate environmental change and induces the expression of a group of genes that permit adaptation to the altered environment [2].

ECF (Extra-cytoplasmic function) factors, as group IV sigma, have two domains, σ2 and σ4, which are responsible for the direct recognition of -10 and -35 elements in the promoter, respectively [1, 3–6]. SigW is one of ECF sigma factors in the gram-positive model bacterium *Bacillus subtilis*. Its activity is suppressed by the anti-sigma RsiW under normal growth conditions [7, 8]. The expression of the SigW regulon is induced in response to diverse envelope stresses, such as antimicrobial reagents [9, 10], alkaline shock [11], salt shock [12], and phage infection [11].

Anti-sigma RsiW is a single transmembrane protein that has a cytoplasmic and an extra-cellular domain. The cytoplasmic domain (RsiW_cyto_) interacts tightly with SigW to inhibit SigW activity [8]. Under stress conditions, the transmembrane segment of RsiW is cleaved sequentially by the membrane proteases, PrsW and RasP (YluC). RsiW fragment that binds to SigW is then destroyed by ClpXP protease [7, 13, 14], resulting in SigW activation, followed by the elevated expression of its regulon. The sequential cleavage of RsiW is similar to the regulated intramembrane proteolysis (RIP) of *E*. *coli* RseA by DegS and RseP proteases [15–17]. RIP of RsiW implies that the destruction of anti-sigma RsiW mediates the transfer of the envelope stress signals to the cytoplasm across the plasma membrane to activate SigW.

RsiW is classified as a ZAS family protein. Its cytoplasmic domain contains the C_n_HX_3_CX_2_C motif for the coordination of a zinc ion [18]. Some ZAS family proteins regulate the activity of a cognate sigma factor through the zinc-binding motif. For example, the cytoplasmic anti-sigma RsrA from *S*. *coelicolor* discharges SigR in response to oxidative stresses [19–21]. The conformational change of RsrA caused by the oxidation of the zinc-binding motif results in SigR release, followed by the increased expression of the SigR regulon [22, 23]. Recently, it was suggested that the first cysteine in the CHCC motif functions as the sensor of oxidative stresses and induces a conformational change by forming a disulfide bond with either of the other two cysteines [23]. Although RsiW has a CHCC motif for zinc coordination like RsrA, its known functions suggest that RsiW activity for SigW suppression is regulated by the RIP of RsiW rather than by the state of zinc coordination.

To understand the regulation mode of SigW activity by RsiW, we determined the crystal structures of SigW/RsiW_cyto_ complexes under reduced and oxidized conditions. In the structure, RsiW_cyto_ interacts with the groove formed along the surface of two domains in SigW (σ^W^_2_ and σ^W^_4_), resulting in the burial of the promoter binding area of SigW. Notably, SigW and RsiW_cyto_ were co-purified in a zinc free oxidation state. The structure of the oxidized CHCC motif in RsiW_cyto_ shows a disulfide bond between the first (C3) and third cysteines (C37). The overall conformation and binding interface between SigW and RsiW were not changed by oxidation. Thus, the data suggest that the CHCC motif in RsiW is not a sensor for oxidative stresses.

## Materials and methods

### Plasmid preparation, protein expression and purification

DNA encoding SigW (residues 1-187) and RsiW_cyto_ (residues 1-80) were amplified from the genome of *B*. *subtilis* 168 strain by PCR. DNA fragments encoding His_x6_-thioredoxin-RsiW_cyto_ and SigW were inserted into the MCS1 and MCS2 of pET-DUET1 vector (Novagen), respectively. The plasmid was then transformed into *E*. *coli* strain BL21-star (DE3) (Invitrogen) and the cells were grown in LB medium. Protein expression was induced using 0.4 mM IPTG at 20°C. After overnight induction, the cells were harvested by centrifugation and the clarified cell lysates were prepared in buffer A (20 mM HEPES pH 7.5, 0.2 M NaCl, 5% glycerol, 5 μM ZnCl_2_, and 0.5 mM TCEP). The complex of SigW and RsiW_cyto_ was purified by immobilized metal affinity chromatography (IMAC) and size exclusion chromatography (SEC). The proteins purified by IMAC were treated with TEV protease to remove the His_x6_-thioredoxin tag from RsiW_cyto_. After complete cleavage of the tag, the protein solution was dialyzed in buffer A. After dialysis, the protein solution was passed through IMAC resin to remove His_6x_-thioredoxin from the protein solution. The complex was further purified by Superdex75 size exclusion chromatography (GE Healthcare) in buffer A. The purified protein was concentrated to 20 mg/mL and estimated to be >95% purity by SDS-PAGE. To purify the zinc-free oxidized complex, SigW and RsiW_cyto_ were co-expressed in minimal media that did not contain zinc, and were purified by the same procedures as the zinc binding complex, using buffer B (20 mM HEPES pH 7.5, 0.2 M NaCl, and 5% glycerol). For the expression and purification of the seleno-methionine labeled complex, the plasmid was transformed into *E*. *coli* strain B834 (DE3) (Novagen) and the cells were grown in minimal media containing Se-met. The complex was purified in the same buffer condition as the zinc binding complex.

### Crystallization and data collection

The crystallization of SigW/RsiW_cyto_ complex was performed using the micro-batch method at 20°C. The crystallization drop was prepared by mixing 1 μL of protein solution (15 mg/ml) and 1 μL of crystallization solution (0.1 M HEPES pH 7.5, 17% (v/v) PEG3350, 3% (v/v) isopropanol, and 0.1 M CaCl_2_) under a layer of Al’s oil (Hampton Research). Crystals of SigW/RsiW_cyto_ complex were fully grown in two weeks. Crystals were picked up with a cryo-loop (Hampton Research) after adding 0.3 μL of 100% glycerol directly to a crystallization drop and were flash-frozen in a cold nitrogen stream. The diffraction data were collected at PLS-BL7A (Beam line 7A, Pohang Light Source, South Korea) and were indexed, integrated, and scaled using HKL2000 [24]. The diffraction data of the Se-Met derivative were collected at two wavelengths (peak and remote) (Table 1).

**Table 1:** Data Collection and Refinement Statistics.

Data collection					
Data set		SigW/RsiWcyto (+Zn^2+^)	SigW/RsiWcyto (-Zn^2+^)	MAD data	
				Peak	Edge
Space group		P2_1_2_1_2_1_			
Unit cell					
	a, b, c (Å)	63.46, 64.21, 138.37	63.08, 63.52, 138.55	62.03, 63.76, 137.57	
	α, β, γ (°)	90.00, 90.00, 90.00	90.00, 90.00, 90.00	90.00, 90.00, 90.00	
Resolution (Å)		30.0-2.8 (2.85-2.80)	30.0 – 2.6 (2.64-2.60)	30.0-2.8 (2.85-2.80)	
Wavelength (Å)		0.97933	0.97926	0.97917	0.97142
Total/Unique reflections		80013/14411	123669/17578	95759/26114	94615/25968
Completeness (%)		98.9 (100.0)	99.1 (100.0)	99.5 (99.7)	99.5 (99.1)
I/σ		41.5 (5.7)	46.3 (6.4)	28.3 (1.6)	24.3 (1.5)
Rmerge (%)		7.0 (44.5)	6.0 (42.1)	7.5 (79.7)	7.3 (82.6)
Figure of merit				0.430	
**Refinement**					
Resolution (Å)		30.0-2.8	30.0-2.6		
No. reflections, working/free		13669/662	16625/896		
Rwork/Rfree (%)		21.0/28.7	23.7/26.8		
No. atoms					
	Protein	3734	3726		
	Zn^2+^	2	0		
B factors		85.0	82.3		
RMSD					
	Bond length (Å)	0.009	0.009		
	Bond angle (°)	1.289	1.311		
Ramachandran plot (%)					
	Favor	98.4	98.0		
	Allowed	1.6	1.8		
	Disallowed	0.0	0.2		

### Structure determination and refinement

The crystal structure of the SigW/RsiW_cyto_ complex was determined by the MAD method. The initial experimental map and a partial model were calculated in PHENIX [25] and manual model building was performed using COOT [26]. Cycles of refinement and model rebuilding were performed at 2.9 Å resolution using PHENIX.refine [27] and COOT programs. The structures of the zinc-binding and oxidized forms were refined and rebuilt at 2.8 and 2.6 Å resolutions after molecular replacement using the model of se-Met derivative as a template [28]. Final rounds of refinement were performed using REFMAC with TLS restraint [29, 30]. The final structures of the zinc-coordinating and oxidized forms were refined with R/R_free_ (%) values of 21.0/28.7 and 23.7/26.8, respectively, and evaluated using PROCHECK [31], which revealed that no residue fell in the disallowed region of the Ramachandran plot, except for Gly15 in the chain B of the oxidized form. The data collection and refinement statistics are summarized in Table 1. The figures were drawn using PyMOL [32, 33]. The surface area, protein-protein interaction and structural alignment were analyzed using PISA [34], DIMPLOT [35], and the DALI server [36], respectively.

### Size exclusion chromatography with multi-angle laser light scattering (SEC-MALLS)

The purified SigW/RsiW_cyto_ complex was injected into a Superdex200 analytical column with Purifier FPLC (GE Healthcare) and the elution products were applied to inline DAWN HELEOS MALLS and Optilab rEX differential refractive index detectors (Wyatt Technology Corporation). Data were analyzed with the ASTRA V software package (Wyatt Technology Corporation).

## Results

### Structure determination of *B*.*subtilis* SigW/RsiW_cyto_

**Fig 1:**
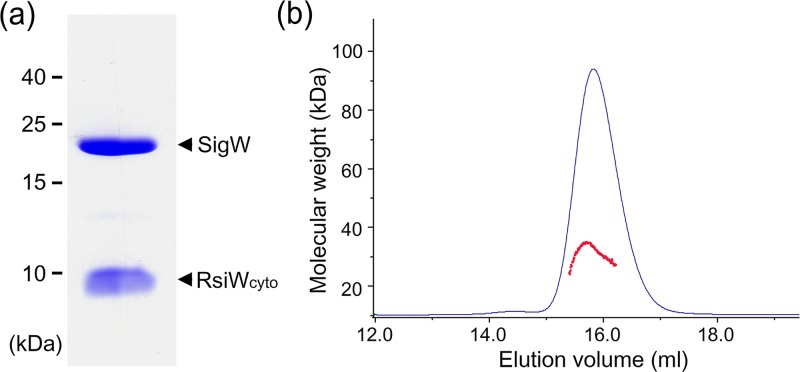
Characterization of the SigW/RsiW_cyto_ complex. (a) SDS-PAGE of SigW/RsiW_cyto_. SigW and RsiW_cyto_ were co-eluted in all the purification steps. (b) SEC-MALLS measurement. The purified SigW/RsiW_cyto_ is a monodisperse complex with single copy stoichiometry of each subunit.

Recombinant SigW and a cytoplasmic domain of RsiW (RsiW_cyto_) were co-expressed in *E*. *coli* and co-purified by metal affinity and size exclusion chromatography (Fig 1A). The molecular weight of the complex, as calculated by size exclusion chromatography-multiangle laser light scattering (SEC-MALLS), was approximately 31.5 kDa (Fig 1B), indicating that the SigW/RsiW_cyto_ complex is a hetero-dimer in solution. Rod-shaped crystals grown in the condition containing polyethylene-glycol 3350 as a precipitant diffracted up to 2.8 Å resolution (S1 Fig). Although SigW shares high sequence identity with *E*. *coli* SigE (36.6% sequence identity), structure determination by molecular replacement was not possible, indicating that the conformation of SigW may be different compared with the structures of other group IV sigma factors. Thus, the structure was determined by the multi-wavelength anomalous dispersion (MAD) method, using a crystal of selenomethionine-labeled SigW/RsiW_cyto_. The residues of two SigW/RsiW_cyto_ complexes in the asymmetric unit (residues 3-95 and 125-187 in SigW and residues 3-72 in RsiW) were traced into the electron density and the final model was refined with R/R_free_ values of 21.0/28.7% (Table 1). Two SigW/RsiW_cyto_ complexes in the asymmetric unit are superimposed with a root-mean-square deviation (rmsd) of 0.7 Å for 224 Cα atoms, indicating that the two complexes have the same conformation.

### Overall structure of *B*. *subtilis* SigW/RsiW_cyto_

**Fig 2:**
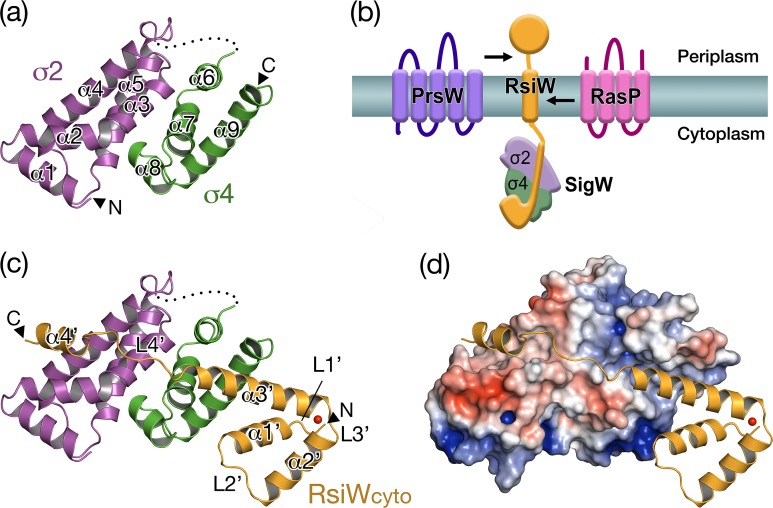
The structure of SigW/RsiW_cyto_ complex. (a) The ribbon model of SigW. σ^W^_2_ (magenta; residues 3-95) and σ^W^_4_ (green; residues 125-187) form globular folds that consist of five (α1-α5) and four alpha-helices (α6-α9), respectively. The N-terminus and C-terminus are labeled to N and C. (b) The model figure that shows SigW inhibition by RsiW. The cytoplasmic domain of RsiW binds to SigW. RsiW is destroyed sequentially by two membrane proteases, PrsW (purple) and RasP (magenta), in response to extracellular stress signals. (c) The ribbon model of the SigW/RsiW_cyto_ complex. RsiW_cyto_ (orange, residues 3-72) consists of four alpha-helices (α1’-α4’) and linkers (L1’-L4’). α1’-α3’ form a helical bundle that coordinates a zinc ion (red sphere) and interact with σ^W^_2._ L4’-α4’ interact with σ^W^_2_. (d) Surface binding model. The α3’-L4’-α4’ of RsiW_cyto_ are fitted into the relative hydrophobic surface groove on SigW. The C-terminus of α1’ binds to a positively charged surface on SigW.

SigW comprises N-terminal σ2 (σ^W^_2_) and C-terminal σ4 (σ^W^_4_) domains, each of which forms a canonical fold in group IV sigma factors. The two domains are connected by a flexible loop (residues 96-125) that is fully disordered. Both σ^W^_2_ (residues 3-95) and σ^W^_4_ (residues 125-187) form alpha-helical folds that contain five (α1-α5) and four alpha-helices (α6-α9), respectively (Fig 2A). σ^W^_2_ is superimposed on the σ^E^_2_ in *E*. *coli* SigE [37] with an rmsd value of 1.4 Å for 88 Cα positions, and σ^W^_4_ is superimposed on the σ^E^_4_ with an rmsd value of 1.8 Å for 64 Cα positions. SigW forms a compact globular structure by the interaction between σ^W^_2_ and σ^W^_4_ (Fig 2). Although the folds of σ^W^_2_ and σ^W^_4_ are similar to those of *E*. *coli* SigE, full-length SigW does not align with SigE, suggesting that two domains in group IV sigma factors can be arranged in diverse conformations.

RsiW_cyto_ contains four alpha-helices (α1’-α4’). The three N-terminal alpha-helices (α1’-α3’) form a helical bundle and coordinate a zinc ion. To confirm the zinc coordination, SigW/RsiW_cyto_ was co-expressed in LB-media containing trace amounts of zinc ion. It was purified and crystallized in the absence of zinc ions. The x-ray emission spectrum measured in the synchrotron facility revealed that the crystal contains zinc ion bound to SigW/RsiW_cyto_ (S2 Fig). In the structure of RsiW_cyto_, the zinc ion is coordinated by residues C3 in L1’, H30 in α2’, C34 in L3’, and C37 in α3’ (Fig 2).

In addition to the three N-terminal helices, the C-terminal α4’ is connected to α3’ through a linker sequence (L4’). The secondary structure of RsiW_cyto_ appears to be similar to *E*. *coli* RseA_cyto_, which comprises four alpha-helices. However, they do not share the same tertiary structure. Taken together, SigW/RsiW_cyto_ and *E*.*coli* SigE/RseA_cyto_ show different domain arrangements and interaction modes because of domain flexibility, even though they share similar secondary structures and domain folds.

### Interaction between SigW and RsiW_cyto_

**Fig 3:**
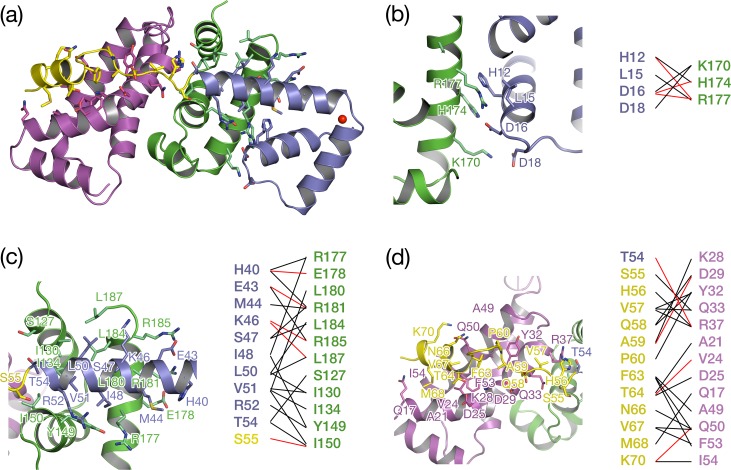
Interaction between SigW and RsiW_cyto_. (a) The ribbon and stick model of SigW/RsiWcyto. σ^W^_2_ (magenta) and σ^W^_4_ (green) interact with α1’-α3’ (purple) and L4’-α4’ (yellow) of RsiW, respectively. The stick model indicates the residues in the binding interface and the rectangular shapes colored black, red, and blue, indicate the three binding interfaces shown in (b), (c), and (d), respectively. (b-d). Three binding interfaces. The residues that mediate the interaction are drawn as a stick model and labeled. The left parts in each figure show direct interactions between SigW and RsiW. Black and red lines between two residues indicate a hydrophobic interaction and a hydrogen bond (or ionic interaction), respectively.

In the structure of the *E*. *coli* SigE/RseA_cyto_ complex, RseA_cyto_ is sandwiched and its N-terminal helical bundle is buried between σ^E^_2_ and σ^E^_4_ [37], providing a large binding surface area (5561 Å^2^). By contrast, RsiW_cyto_ interacts with SigW by wrapping the surface of SigW, resulting in a large surface burial of 3324 Å^2^ (Fig 2D). The surface binding area of SigW/RsiW_cyto_ is divided into three binding motifs of RsiW_cyto_ (Fig 3A). Binding motif 1, containing residues H12, L15, D16, and D18 located on α1’ and L2’ in RsiW_cyto_, interacts with the positively charged surface formed by residues K170, H174, and R177 in the σ4 domain of SigW, resulting in a surface burial of 489 Å^2^ (Fig 3B). D16 in binding motif 1 is in a central position that mediates an ionic interaction and a hydrogen bond with K174 and R177 in SigW. Binding motifs 2 and 3 are fitted into a long hydrophobic groove, perpendicular to the interaction surface between σ^W^_2_ and σ^W^_4_ (Figs 2D and 3A). Binding motif 2 is located mainly on the C-terminal half of α3’ in RsiW_cyto_, which is fitted along the groove formed by residues of σ^W^_4_ (Fig 3A and 3C), and binding motif 3, which contains residues on α3’ and L4’, binds to the groove formed by residues in σ^W^_2_ (Fig 3A and 3D). The interaction between the surface groove on SigW and binding motifs 2 and 3 in RsiW_cyto_ results in surface burials of 1316 and 1805 Å^2^, respectively.

### Inhibition of promoter recognition of SigW by RsiW

**Fig 4:**
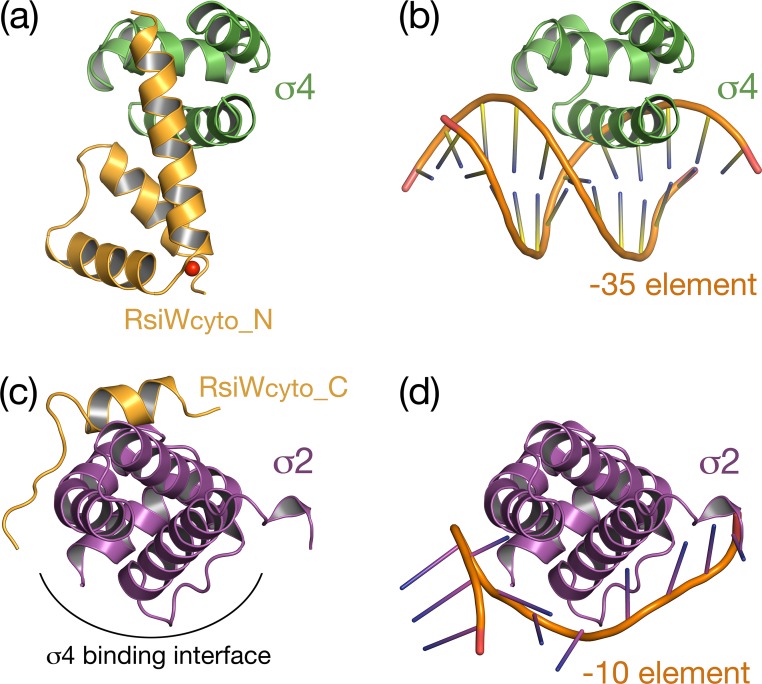
Inhibition mode of promoter recognition of SigW by RsiW. (a) The ribbon model showing the interaction between σ^W^_4_ and RsiW. (b) The model of -35 element recognition by σ^W^_4_, generated by the superimposition with the structure of *E*.*coli* σ^E^_4_/-35 element (PDB ID: **2H27**). (a, b) σ^W^_4_ ribbon models are drawn in the same orientation. α1’ in RsiW interacts directly with the recognition helix for the -35 element binding. The N-terminal helical bundle (α1’-α3’) of RsiW sterically restricts the DNA recognition of SigW. (c) The ribbon model showing the interaction between σ^W^_2_ and RsiW. (d) The model of the -10 element recognition by σ^W^_2_, generated by the superimposition with the structure of *T*. *acuaticus* σ2/single strand -10 element (PDB ID: **3UGO**). (c, d) The σ^W^_2_ surface for the recognition of the -10 element is blocked by σ^W^_4_ binding in the SigW/RsiW complex, rather than being blocked directly by RsiW.

The σ2 and σ4 domains of group IV sigma factors interact with -10 and -35 elements in the promoter, respectively, to initiate transcription of its regulon. The structure of *E*. *coli* σ^E^_4_/-35 element reveals that the recognition helix of the σ^E^_4_ helix-turn-helix motif, which corresponds to α9 in SigW, binds to the major groove of -35 elements [5]. Thus, the direct interaction between the residues on α9 of SigW (K170, H174 and R177) and α1 in RsiW implies that RsiW restricts σ^W^_4_ binding to the -35 element directly (Figs 3B and 4A). The model structure of the σ^W^_4_/-35 element generated by the superimposition of *E*. *coli* σ^E^_4_/-35 and σ^W^_4_ shows that the binding of RsiW and the -35 element to SigW is mutually exclusive (Fig 4B). Whereas, in the model structure of σ^W^_2_/single strand -10 element prepared by superimposition of σ^W^_2_ and *T*. *aquaticus* σ^A^_2_/single strand -10 element [6], the distinct σ^W^_2_ surfaces mediate RsiW and -10 motif binding, implying that RsiW does not inhibit the binding of σ^W^_2_ to the -10 motif directly. Instead, the binding surface of the -10 element is buried by σ^W^_4_ in the SigW/RsiW_cyto_ structure (Fig 4C and 4D). Taken together, anti-sigma RsiW inhibits the promoter binding of SigW by directly blocking the binding surface of -35 element in σ^W^_4_. Additionally, the binding of -10 element to σ^W^_2_ is also inhibited by the domain arrangement of SigW.

### Comparison of zinc coordination motifs between RsiW^Red/Zn2+^ and RsiW^ox^

RsiW has been classified as a member of the ZAS subfamily, based on sequence alignments [18]. One of the ZAS proteins, *S*. *coelicolor* RsrA, releases a zinc ion in response to oxidative stresses and activates SigR by reducing SigR binding affinity. Both RsiW and RsrA contain a CHCC motif for zinc coordination. It is inferred that zinc binding by RsiW might be also involved in the regulation of SigW activity by changing SigW binding affinity. However, SigW regulation by oxidative stresses has not been observed. SigW activity has been demonstrated to be regulated by the RIP of RsiW in response to extra-cytoplasmic stresses.

**Fig 5:**
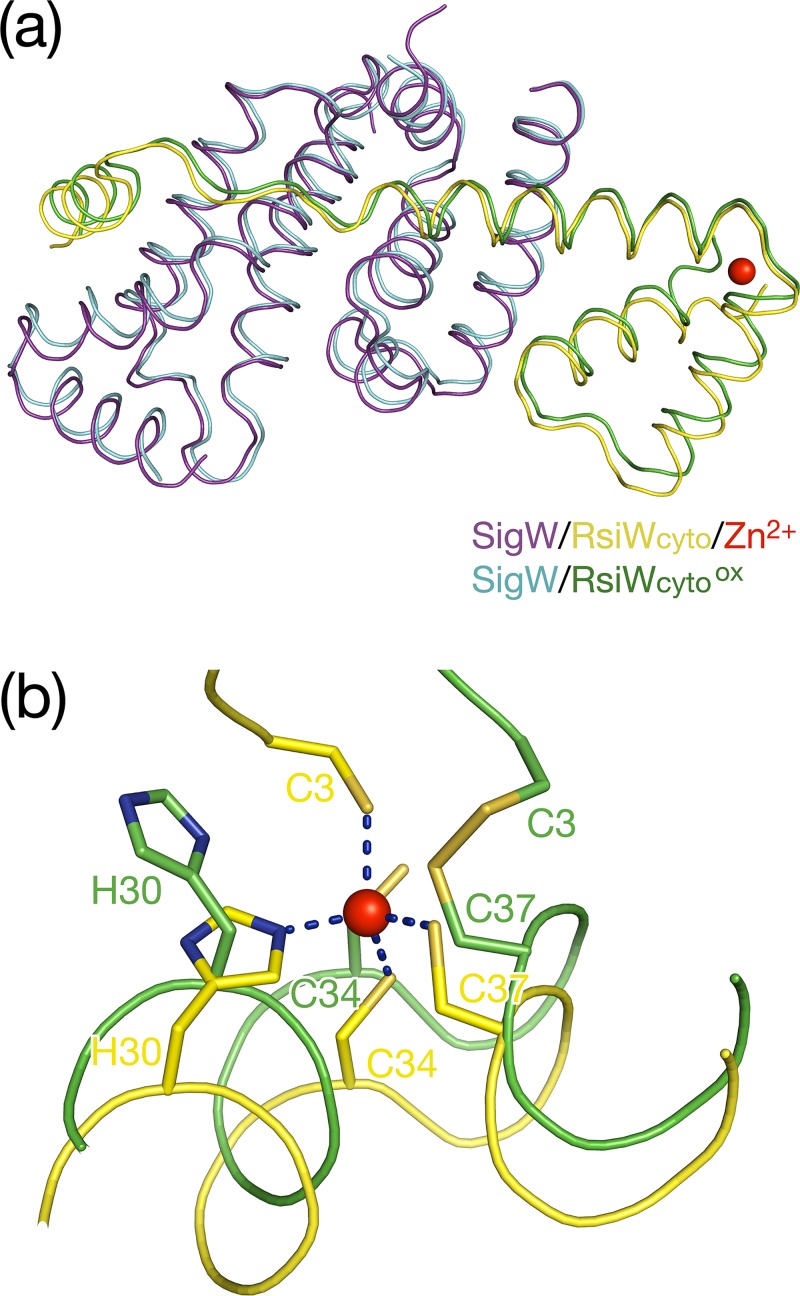
The structures of SigW/RsiW_cyto_ in the zinc-binding and oxidation states. (a) The superposition of Cα traces of SigW/RsiW_cyto_^Red/Zn2+^ (zinc binding complex; purple and yellow) and SigW/RsiW_cyto_^ox^ (the complex in oxidaized state; teal and green). SigW and RsiW_cyto_ interact with each other, maintaining the same overall conformation in both states. The absence of zinc does not induce a conformational change. (b) Comparison of the CHCC motifs. Residues C3, H30, C34, and C37 coordinate a zinc ion in reduced state (yellow stick model). Residues C3 and C37 form a disulfide bond in oxidized state (green stick model).

In the crystal structure of SigW/RsiW_cyto_, a helical bundle at the N-terminus coordinates a zinc ion through residues C3, H30, C34, and C37 (Fig 5). To confirm whether RsiW_cyto_ still binds to SigW in the absence of zinc ions, SigW and RsiW_cyto_ were co-expressed in minimal media that did not contain zinc ions and were co-purified in the absence of a reducing agent and zinc ions to oxidize the zinc coordination motif (SigW/RsiW_cyto_^ox^). Interestingly, RsiW_cyto_ was co-eluted with SigW during purification, and the protein properties observed were same as a zinc binding complex (SigW/ RsiW_cyto_^Red/Zn2+^). SigW/RsiW_cyto_^ox^ purified in zinc-free oxidation state was crystallized at the same condition as SigW/ RsiW_cyto_^Red/Zn2+^, and the structure was determined by molecular replacement using the structure of SigW/RsiW_cyto_^Red/Zn2+^ as a template. The final model was refined with R/R_free_ values of 23.7/26.8% (Table 1). Two structures of SigW/RsiW_cyto_ in the zinc-bound and zinc-free states are superposed with an rmsd of 0.8 Å for 223 Cα atoms. This reveals that the absence of zinc ion does not change the overall conformation of SigW/RsiW_cyto_ (Fig 5A).

RsiW_cyto_^Red/Zn2+^ and RsiW_cyto_^ox^ overlap with an rmsd of 1.0 Å for 69 Cα atoms. The major difference between the two structures resides in the zinc-binding motif. The structure of SigW/RsiW_cyto_^ox^ clearly shows that a disulfide bond is formed between C3 and C37. In addition, H30 and C34 recede from the positions required for zinc coordination (Fig 5B and S3 Fig). Although there is a slight conformational change induced by the absence of zinc and oxidation, the interaction between SigW and RsiW_cyto_ is not changed. All the residues in the three SigW binding motifs of RsiW_cyto_ (Fig 3) participate in SigW binding, resulting in a surface burial of 3306 Å^2^. In terms of the binding surface area and the number of bonds between SigW and RsiW_cyto_, zinc coordination of RsiW does not appear to change its ability to inhibit SigW, suggesting that SigW is not activated by oxidation or the absence of zinc in the CHCC motif of RsiW.

### Comparison of binding modes between the group IV sigma and cognate anti-sigma factors

**Fig 6:**
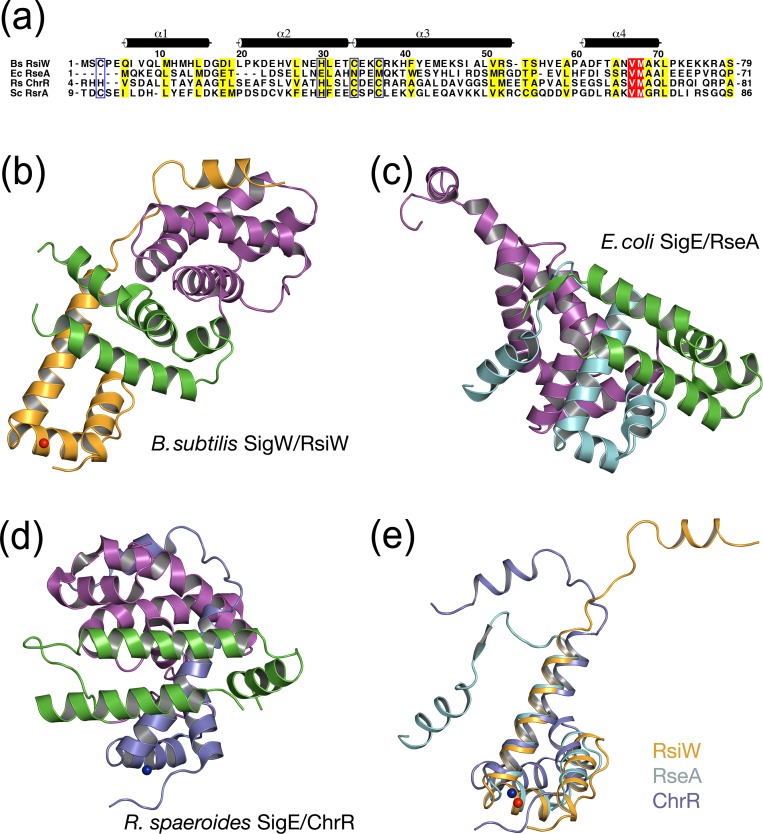
Structural comparison of group IV sigma/anti-sigma factors. (a) Sequence alignment of anti-sigma factors. Species abbreviations are as follows: Bs, *B*. *subtilis*; Ec, *E*.*coli*; Rs, *R*. *sphaeroides*; Sc, *S*. *coelicolor*. The residues aligned with a CHCC in Bs RsiW are marked with blue boxes. Coordinates of Ec RseA and Rs ChrR used in the structure alignment were obtained from Protein Data Bank (PDB IDs: 1OR7 and 2Z2S). (b-d) Ribbon models of the complexes. σ2 and σ4 in sigma factors are colored magenta and green, respectively. Anti-sigma domains of RsiW (b), RseA (c), and ChrR (d) are colored orange, teal, and purple, respectively. The models are drawn in the same orientation after N-terminal helical bundles (α1’-α3’) of anti-sigma are superimposed. (e) The superimposition of anti-sigma domains. The positions of the C-terminal motifs are variable, depending on the binding of the cognate sigma factor.

Although there is sequence diversity among group IV anti-sigma factors, the secondary structures of sigma-binding domains in anti-sigma proteins are conserved as four alpha-helices [18]. In the comparison of the three anti-sigma domains, RsiW_cyto_, *E*. *coli* RseA_cyto_, and *R*. *sphaeroides* ChrR-ASD, the folds formed by the first three alpha helices (α1’-α3’) share similar conformations, regardless of the presence or absence of a zinc binding motif (Fig 6). Meanwhile, the N-terminal helical bundle in *E*. *coli* RseA and *R*. *sphaeroides* ChrR mediates the binding of both σ2 and σ4 domains by being sandwiched between the domains, the α1’-α3’ in RsiW interacts only with σ4 (Fig 6B–6D). To confirm whether the interface between σ2 in SigW and the α1’-α3’ motif in RsiW_cyto_ can be stabilized by the interaction, σ^W^_2_ and α1’-α3’ in SigW/RsiW_cyto_ were superimposed onto the structure of *E*. *coli* SigE/RseA_cyto_. The binding interface shows a few charge repulsions (D18-E32, D25-E45, and K30-R52 in σ2/α1’-α3’) and contacts between charged and hydrophobic residues (D22/L31 and M4-H14). This suggests that the conformation of SigW/RsiW_cyto_ is not an artifact of crystal packaging but appears to be an intrinsic structure.

The α4’ interacts with σ^W^_2_ in the structures of SigW/RsiW_cyto_, *E*. *coli* SigE/RseA_cyto_, and *R*. *sphaeroides* SigE/ChrR-ASD. The relative positions of α4’ are variable among the anti-sigma structures upon which the α1’-α3’ motifs are superimposed (Fig 6E). This suggests that L4’ and α4’ in RsiW are a flexible motif that interacts with σ^W^_2_, regardless of the arrangement between σ2 and σ4.

## Discussion

Anti-sigma RsiW in *B*. *subtilis* inhibits the SigW activity required for the transcription initiation of stress response genes [7, 8]. The cytoplasmic anti-sigma domain in RsiW has a CHCC motif for zinc coordination [18, 38], as a ZAS family protein that may be involved in the activity regulation of sigma factor through zinc coordination. Although some ZAS factors recognize oxidative stresses and regulate the activities of their cognate sigma factors through the CHCC motif [19–22], the inhibitory activity of a single transmembrane RsiW has been shown to be regulated by RIP by two membrane proteases, PrsW and RasP, [7, 13, 39] and to be insensitive to oxidative stresses [22]. In this study, we determined the first structure of a sigma/ZAS factor complex in the oxidized state. The crystal structures of the SigW/RsiW_cyto_ complex in the reduction/zinc binding and oxidation states explain the zinc coordination mode of RsiW, as well as the inhibition mode of SigW activity by RsiW.

To inhibit the promoter binding of SigW, RsiW interacts with the SigW surface groove (Fig 2). The -35 element binding surface of SigW is blocked by the direct binding of RsiW and the -10 element binding surface is buried into σ^W^_4_ binding interface under RsiW binding (Fig 4). The RsiW binding mode to SigW is different from other group IV anti-sigma factors that have four helices for sigma binding. *E*. *coli* RseA and *R*. *sphaeroides* ChrR are sandwiched between cognate σ2 and σ4 domains, while RsiW binds to SigW surface (Fig 6). *C*. *metallidurans* CnrY, which has a short sigma binding motif corresponding to the α3’ and α4’ of RsiW, shows a similar sigma binding mode to RsiW, even though CnrY does not have the two N-terminal helices required for zinc coordination and the additional interaction with sigma factor. It suggests that the zinc binding motif of RsiW is not a major factor that determines the conformation of SigW/RsiW_cyto_. In this regard, the inhibition mode of anti-sigma factors seems to be highly variable. When the domains of SigW/RsiW_cyto_ are superimposed on the sandwiched conformation of *E*. *coli* SigE/RseA_cyto_, the arrangement is not stabilized by the charge repulsion. This suggests that the diverse domain arrangement shown in sigma/anti-sigma structures might be an intrinsic conformation rather than a conformation transformed by crystal packaging.

ZAS family proteins have either a CHCC or a HHCC motif for zinc coordination. *S*. *coelicolor* RsrA that responds to oxidative stresses [19] and RsiW that is insensitive to oxidation have a CHCC motif, while *R*. *sphaeroides* ChrR that responds to singlet oxygen has a HHCC motif [40]. In addition, *E*. *coli* RseA that does not have a zinc coordination motif and RsiW that has an insensitive CHCC motif are regulated by RIP, as ECF anti-sigma factors [16, 17]. This implies that the regulation mode of sigma factor by a cognate anti-sigma factor is not conserved, even though the secondary structures of the sigma binding domain in anti-sigma factors are conserved to 4 helices mostly (Fig 6).

The disulfide bond in the CHCC motif of RsrA results in the dissociation of SigR, allowing SigR to initiate transcription of its regulon including thioredoxins involved in stress responses [19, 41]. In the NMR structure of *S*. *coelicolor* RsrA [23], the disulfide bond between C11 and C44 induces a large conformational change, clustering the hydrophobic residues required for SigR binding (residues V54, L57, V75 and L79) and burying them in hydrophobic core (residues L18, F21, F34 and F38) (Fig 6A). Thus, the oxidized RsrA is excluded from SigR binding. In contrast to RsrA, the binding interface between RsiW and SigW is not disrupted by the formation of a disulfide bond between C3 and C37 (Fig 5). In structure-based sequence alignments (Fig 6A), the hydrophobic residues that induce the conformational change of RsrA in response to oxidation are not fully conserved in RsiW. H14 and S47 in RsiW align with F21 and V54 in RsrA. This suggests that hydrophobic clustering force in RsiW may be not sufficient to induce large conformational change. In mutational studies of RsrA and RsiW [22], the domain swapping of the HCC motif changed the redox sensitivity between RsrA and RsiW and residues flanking the second and third cysteines in the CHCC motif (residues E39, E40, L45 and E46 in RsrA) contributed to redox sensitivity. The three residues in RsrA, except E39, are not conserved in RsiW. Consistent with this data, RsiW_cyto_^ox^ maintains the same overall conformation as RsiW_cyto_^Red/Zn2+^, except for a slight conformational change caused by the oxidation of the zinc binding motif (Fig 5). This supports data showing that the SigW inhibition activity of RsiW is insensitive to oxidative stress or zinc absence.

## Supporting information

S1 Fig The crystal of SigW/RsiW_cyto_. The rod-shaped crystals were obtained by a microbatch method in the condition containing polyethylene-glycol 3350.
(TIF)

S2 Fig X-ray emission spectrum of the SigW/RsiW_cyto_ crystal. The crystal was exposed to excitation energy of 13.0 keV and the emission spectrum shows two peaks at 8.65 and 9.60 keV corresponding to Kα1 and Kβ1 of zinc. The data were collected at PLS-BL7A (Pohang light source, South Korea).
(TIF)

S3 Fig 2F_o_-F_c_ omit electron density maps. The stick models of the CHCC motif in SigW/RsiW_cyto_^Red/Zn2+^ (a) and SigW/RsiW_cyto_^ox^ (b). The omit maps of zinc in (a) and residues C3 and C37 in (b) are drawn at a 1.5 and 1.0 σ contour levels.
(TIF)
