# Supplementary figures and images for: Structural insights into the regulation of Bacillus subtilis SigW activity by anti-sigma RsiW
Source: PLoS One. 2017 Mar 20;12(3):e0174284. doi: 10.1371/journal.pone.0174284 (PMC5358783; doi:10.1371/journal.pone.0174284)

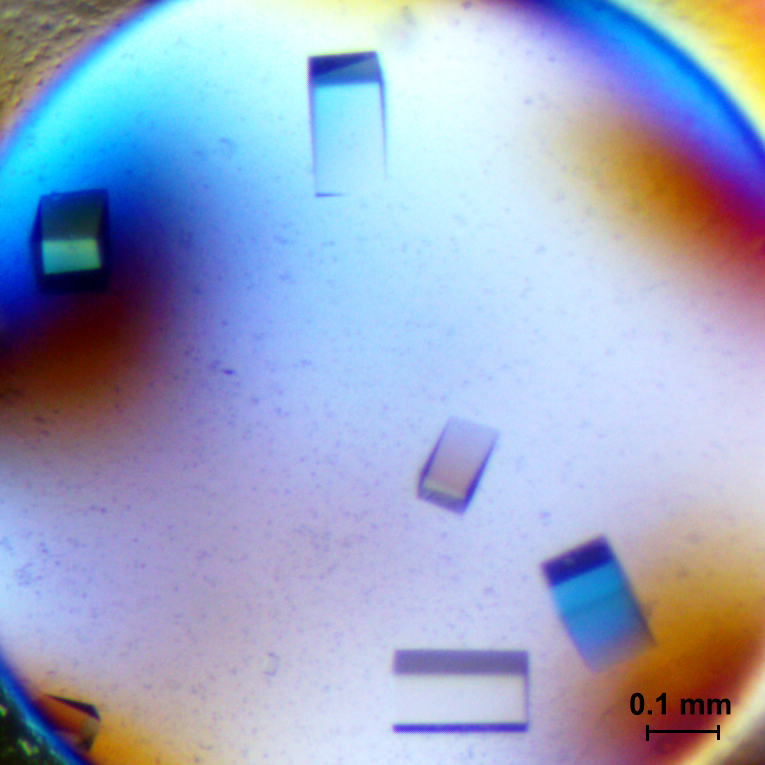

Supplement: S1 Fig — The rod-shaped crystals were obtained by a microbatch method in the condition containing polyethylene-glycol 3350. (TIF) [file pone.0174284.s001.tif]

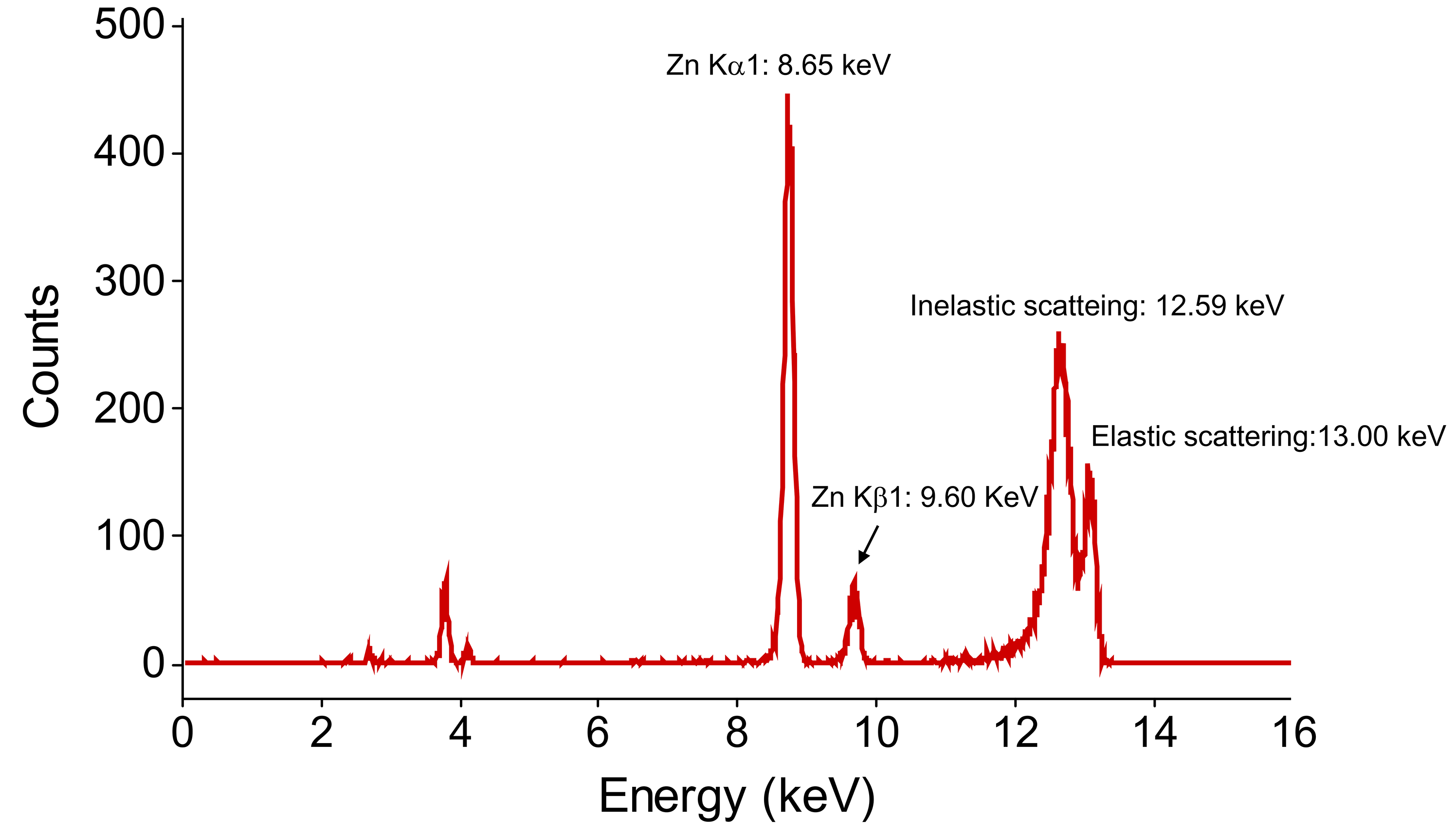

Supplement: S2 Fig — The crystal was exposed to excitation energy of 13.0 keV and the emission spectrum shows two peaks at 8.65 and 9.60 keV corresponding to Kα1 and Kβ1 of zinc. The data were collected at PLS-BL7A (Pohang light source, South Korea). (TIF) [file pone.0174284.s002.tif]

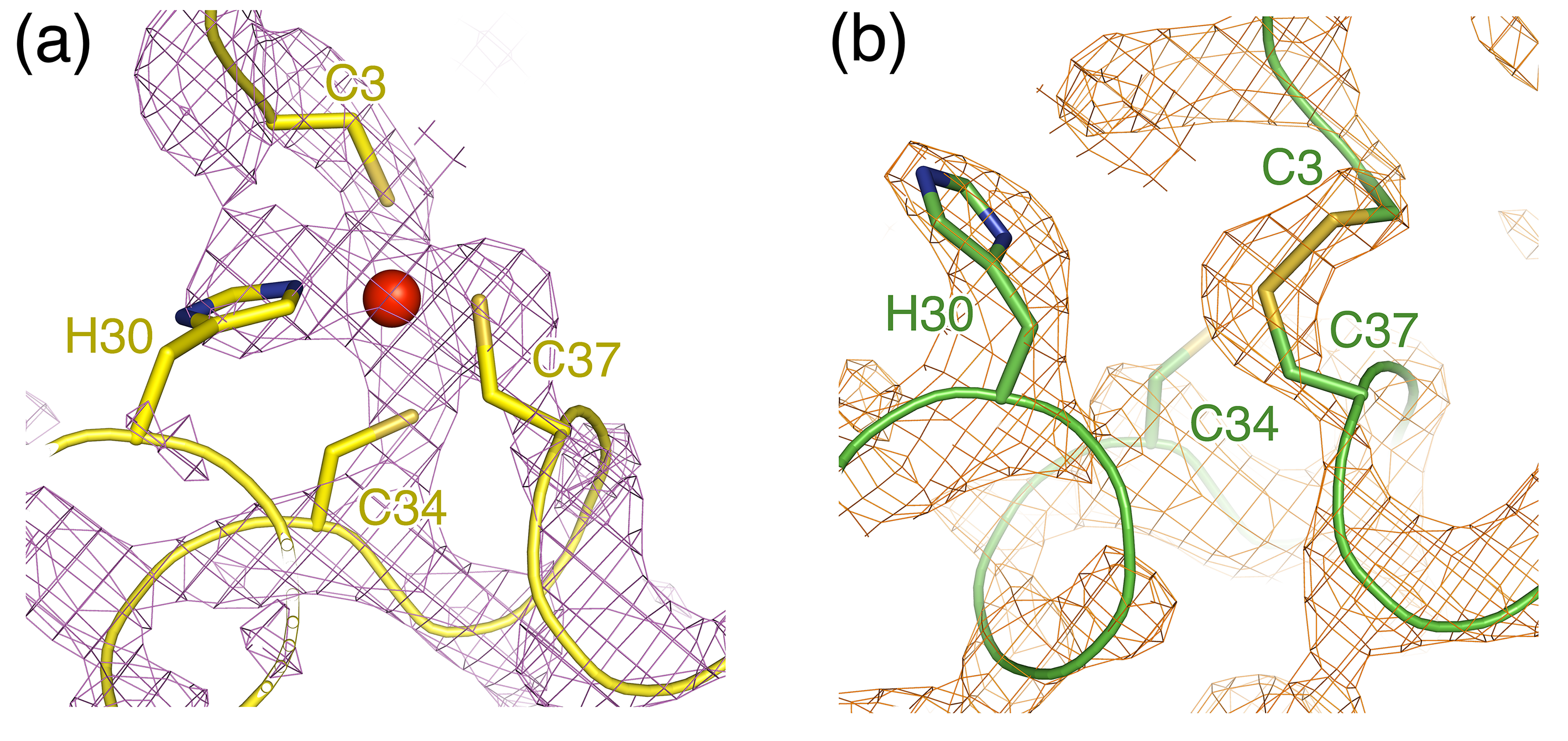

Supplement: S3 Fig — The stick models of the CHCC motif in SigW/RsiWcytoRed/Zn2+ (a) and SigW/RsiWcytoox (b). The omit maps of zinc in (a) and residues C3 and C37 in (b) are drawn at a 1.5 and 1.0 σ contour levels. (TIF) [file pone.0174284.s003.tif]
